# Supplementary material for: Wine Terroir and the Soil Bacteria: An Amplicon Sequencing–Based Assessment of the Barossa Valley and Its Sub-Regions
Source: Front Microbiol. 2021 Jan 7;11:597944. doi: 10.3389/fmicb.2020.597944 (PMC7817890; doi:10.3389/fmicb.2020.597944)
Supplement: Supplementary file 13 [file Image_1.pdf]

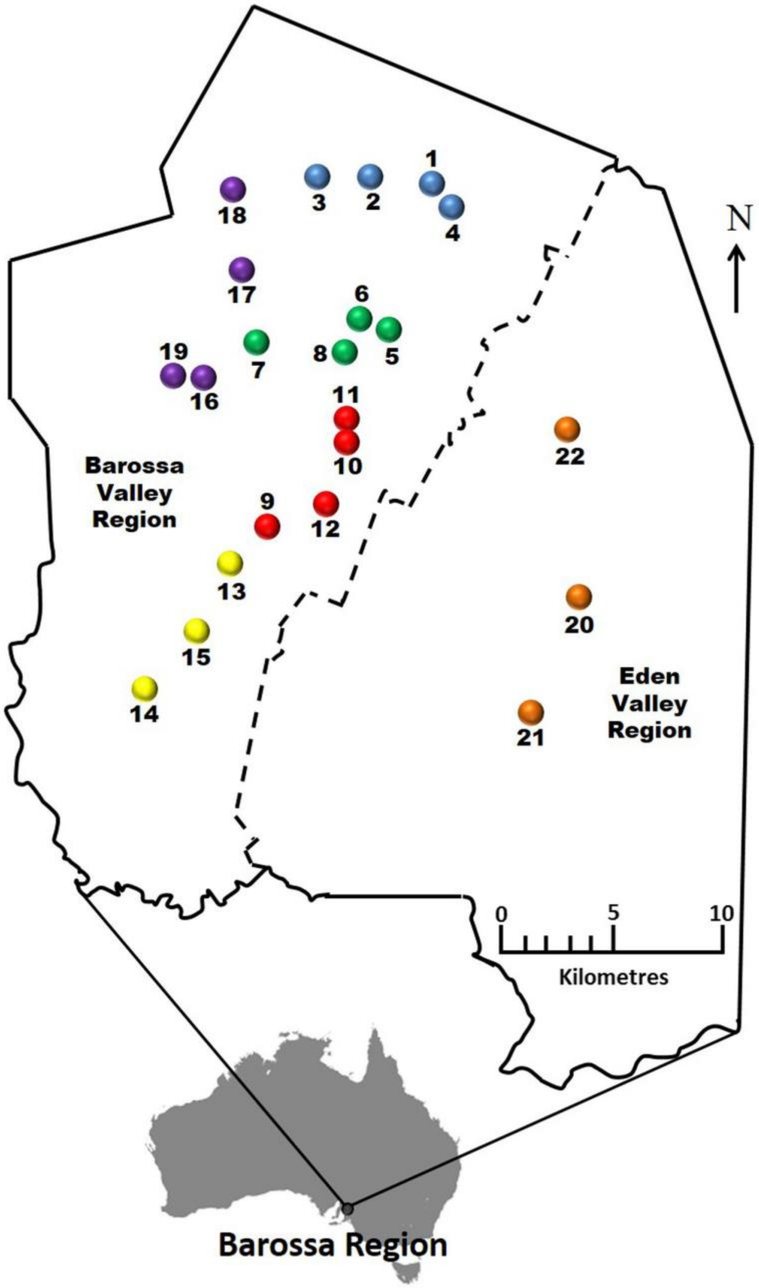

Supplementary Figure 1. Location of 22 Barossa vineyard sites. Vineyards are color coded according to the six wine sub-regions as defined in Xie et al. (2017): Northern Grounds: Blue, Southern Grounds: Yellow, Central Grounds: Green, Eastern Ridge: Red, Western Ridge: Purple, Eden Valley: Orange. Map modified from Xie et al. (2017).
