# Supplementary material for: Wine Terroir and the Soil Bacteria: An Amplicon Sequencing–Based Assessment of the Barossa Valley and Its Sub-Regions
Source: Front Microbiol. 2021 Jan 7;11:597944. doi: 10.3389/fmicb.2020.597944 (PMC7817890; doi:10.3389/fmicb.2020.597944)
Supplement: Supplementary file 15 [file Image_3.pdf]

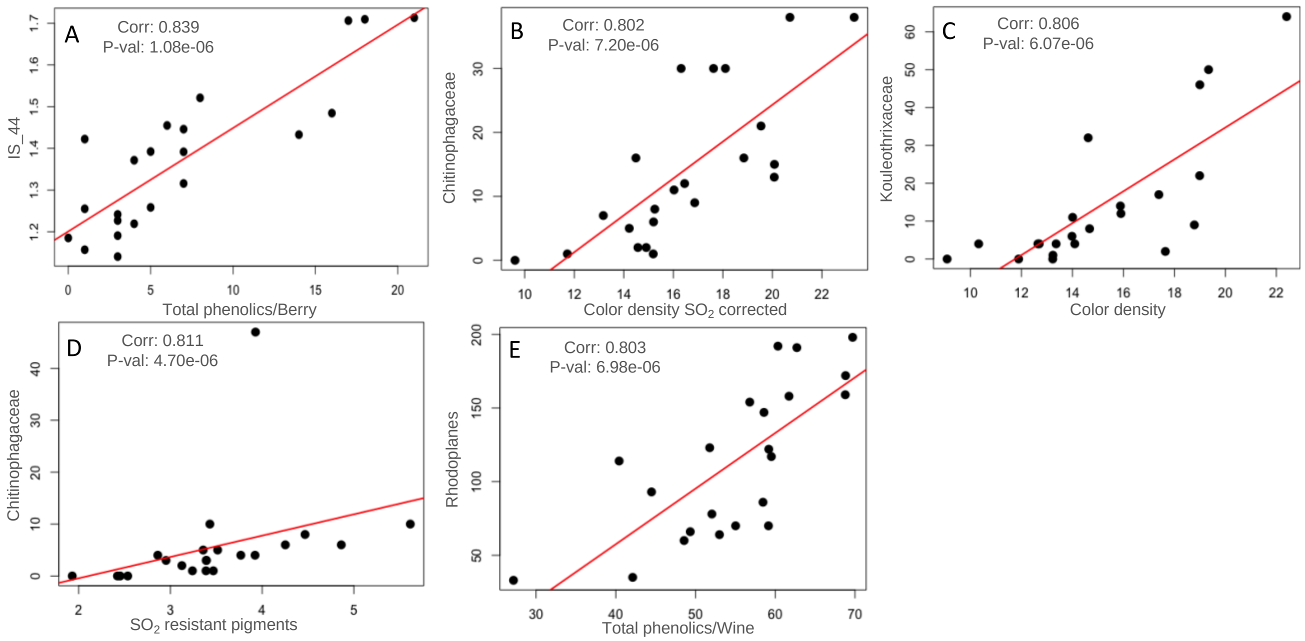

Supplementary Figure 3. Association between taxon abundance and fruit/wine traits in Barossa Region vineyard soil bacteria communities. Relationship between taxon abundance and fruit (A) and wine (B-E) traits. Correlations were tested using Spearman's rank correlation coefficient with its probability estimate for significance (P) and implemented using the function *rcorr* in the R package *Hmisc*. Correlation coefficient and P values for each of the comparisons are included in each inset.
